# Recovery sleep attenuates impairments in working memory following total sleep deprivation

**DOI:** 10.3389/fnins.2023.1056788

**Published:** 2023-04-18

**Authors:** Ziyi Peng, Yanhong Hou, Lin Xu, Haiteng Wang, Shuqing Wu, Tao Song, Yongcong Shao, Yan Yang

**Affiliations:** ^1^School of Psychology, Beijing Sport University, Beijing, China; ^2^Department of Psychology Medical, The 8th Medical Centre of PLA General Hospital, Beijing, China; ^3^State Key Laboratory of Cognitive Neuroscience and Learning, Beijing Normal University, Beijing, China; ^4^IDG/McGovern Institute for Brain Research, Beijing Normal University, Beijing, China; ^5^Center of Stress Medicine, East China Institute of Biotechnology, Peking University, Beijing, China; ^6^Department of Radiology, The 8th Medical Centre of PLA General Hospital, Beijing, China

**Keywords:** sleep deprivation, recovery sleep, working memory, event-related potential, N2, P3

## Abstract

**Introduction:**

The detrimental effects of sleep deprivation (SD) on cognitive function and quality of life are well known, and sleep disturbances are a major physical and mental health issue worldwide. Working memory plays an important role in many complex cognitive processes. Therefore, it is necessary to identify strategies that can effectively counteract the negative effects of SD on working memory.

**Methods:**

In the present study, we utilized event-related potentials (ERPs) to investigate the restorative effects of 8 h of recovery sleep (RS) on working memory impairments induced by total sleep deprivation for 36 h. We analyzed ERP data from 42 healthy male participants who were randomly assigned to two groups. The nocturnal sleep (NS) group completed a 2-back working memory task before and after normal sleep for 8 h. The sleep deprivation (SD) group completed a 2-back working memory task before and after 36 h of total sleep deprivation (TSD) and after 8 h of RS. Electroencephalographic data were recorded during each task.

**Results:**

The N2 and P3 components—which are related to working memory—exhibited low-amplitude and slow-wave characteristics after 36 h of TSD. Additionally, we observed a significant decrease in N2 latency after 8 h of RS. RS also induced significant increases in the amplitude of the P3 component and in the behavioral indicators.

**Discussion:**

Overall, 8 h of RS attenuated the decrease in working memory performance caused by 36 h of TSD. However, the effects of RS appear to be limited.

## 1. Introduction

Proper sleep has been shown to exert beneficial effects on memory, cognitive function, work performance, and immune-related parameters (Walker and Stickgold, 2004; Stickgold, 2005; Anderson and Horne, 2006; Djonlagic et al., 2009). However, the incidence of sleep-related problems continues to increase. While the most direct and obvious behavioral manifestation of sleep deprivation (SD) is drowsiness, it also significantly impairs cognitive function (Drummond et al., 2006; Killgore et al., 2006; Alhola and Polo-Kantola, 2007; Anderson and Platten, 2011). Honn et al. (2020) demonstrated that SD reduces processing speed in visual search, spatial memory, paired associative learning, motor response, and other cognitive tasks. Additional evidence suggests that SD significantly increases response times in working memory tasks. Such changes are also accompanied by decreased activation of the frontoparietal cortex (FPC), which plays an important role in cognitive control. Specifically, the FPC can bypass top-down cognitive control, thus enabling individuals to focus on goal-related information while suppressing irrelevant information (Smallwood et al., 2012; Wen et al., 2013).

Working memory is a limited-capacity system involved in the temporary storage and maintenance of information related to a specific task (Baddeley, 2010). As such, it acts as a bridge between short- and long-term memory (Baddeley, 2000) and is involved in operating, processing, and executing various control processes. Previous studies have consistently demonstrated that SD significantly impairs working memory (Lo et al., 2012; Gerhardsson et al., 2019). In addition to decreasing the quality of information stored in the working memory, SD reduces processing speeds and alters event-related potentials (ERPs) during task performance by prolonging latency and reducing the amplitude of the N2 and P3 components (Zhang et al., 2019). These changes are also associated with a decreased ability to discriminate between target stimuli (Koslowsky and Babkoff, 1992), reduced availability of disposable attentional resources, and alterations in selective attention toward emotional stimuli/signal processing (Schacht et al., 2008).

Numerous research groups have aimed to identify interventions that can effectively counteract the aforementioned negative consequences of SD. Although caffeine and other drugs can effectively maintain work performance and alertness (Rosenthal et al., 1991; Rogers et al., 2005; Biggs et al., 2007), they have limited effects on high-level cognitive functions. In addition, the use of caffeine may be associated with recovery costs in individuals with long-term sleep deficiency (Doty et al., 2017). Recovery sleep (RS) refers to a short period of adequate sleep following SD, and it represents a potential non-pharmacological strategy for combating the effects of SD on cognitive function. Some studies have reported that RS can attenuate SD-induced hyperalgesia (Roehrs et al., 2012; Stroemel-Scheder et al., 2020) and cognitive impairment (Ruggiero and Redeker, 2014). RS also alleviates fatigue and improves attention/alertness, and longer periods of RS are associated with the restoration of cognitive function to a greater degree (Studte et al., 2015). One study reported that 8 h of RS can attenuate impairments in response inhibition caused by 36 h of total sleep deprivation (TSD) (Jin et al., 2015). One night of TSD causes obvious changes in the topological characteristics of the small-world network in the brain. Although two nights of RS can completely restore the global properties of the brain network, it has not been found to induce changes in the local function (Jiang et al., 2018). Following 58 h of wakefulness, sleep stage distribution resembles that of a baseline night when the sleep duration of the recovery night is extended to 14 h (Hennecke et al., 2019). Moreover, a 90-min nap during a day of sleep deprivation can restore hippocampus-dependent learning, and the structural composition of the hippocampus has been shown to predict the success of learning recovery (Saletin et al., 2016). Nevertheless, one or two nights of RS after complete or chronic sleep loss cannot sufficiently attenuate the associated neurobehavioral deficits or restore self-monitoring abilities and brain metabolism (Banks et al., 2010; Lo et al., 2016; Boardman et al., 2018). Therefore, the amount of sleep required to restore cognitive function following extended wakefulness remains unclear.

Impairments in cognitive function due to SD inevitably affect the task performance of individuals. Previous studies have used both neurophysiological and behavioral indicators to assess an individual’s physiological and psychological states. Among these, neurophysiological indicators are more sensitive for assessing the effects of SD. Chai et al. (2020) reported that two nights of RS after one night of TSD restored hippocampal connectivity to normal levels but did not fully restore behavioral performance or its associations with hippocampal connectivity (Chai et al., 2020). A study investigating joint rhythm (Gumenyuk et al., 2014) reported that significant differences in the reorienting negativity amplitude (related to behavioral responses) could be explained by the increased sensitivity of neurophysiological indicators. Electroencephalographic (EEG) data recorded from the human scalp reflect potential changes that indicate the activity of the brain, and ERPs are special evoked potentials related to endogenous neural activity and cognitive function. After RS, information processing efficiency was also restored which related to the promotion and recovery of memory by sleep. The N2 component of the ERP reflects performance monitoring and cognitive control (top-down and bottom-up), and its neural source has most consistently been identified as the anterior midcingulate cortex (aMCC) (Folstein and Van Petten, 2008; Huster et al., 2010). Some researchers have suggested that the P300 component plays a crucial role in brain processing at the intersection between perception and decision making (Verleger et al., 2005). However, others have suggested that it is more commonly involved in synthesizing and carrying information related to conscious access, attentional moderation, and post-response adaptations (Polich, 2007; Dehaene and Changeux, 2011; Raud and Huster, 2017). Previous studies have also demonstrated that the P3 wave is related to the renewal of working memory content and it decreases with an increase in working memory load (Donchin and Fabiani, 1991; Peng et al., 2020). Therefore, N2-P3 components that relate to different information processes will also change after RS (Zhang et al., 2019). Sleep cycles include four stages. Healthy adults need to sleep for 4–5 cycles every night, which typically requires approximately 8 h (Malik et al., 2018). Sleep “reshapes” hippocampal synapses, making room for learning the next day (Spano et al., 2019).

At present, it remains to be determined whether 8 h of RS can attenuate SD-induced impairments in working memory. In the present study, we investigated the potential restorative effects of RS on working memory and ERP latency/amplitude after 36 h of TSD. We tested the following hypotheses: a) 8 h of RS partially attenuates the deleterious effects of 36 h of TSD on working memory in our participants and b) following 36 h of TSD, 8 h of RS reduces the latencies of the working memory-related N2-P3 components and increases their amplitudes. A lack of significant differences between 36 h TSD and subsequent 8 h RS would suggest that the restorative effects of 8 h of RS are limited.

## 2. Materials and methods

### 2.1. Participants

The present study included 42 healthy male college students (mean age, 23 years; age range, 21–28 years). Participants were randomly assigned to two groups: the nocturnal sleep (NS) group (20 participants) and the SD group (22 participants). All participants were right-handed and maintained good sleep habits (Pittsburgh Sleep Quality Index <5 points) (Buysse et al., 1989). None of the patients reported a history of mental or physical illnesses, and none had previously undergone psychophysiological testing. All participants had normal or corrected-to-normal visual acuity, and their IQ scores were greater than the population average (Raven test scores >110 points). The research staff provided a full explanation of the study procedures prior to the experiment. All participants were asked to refrain from smoking, drinking alcohol/coffee, and taking any drugs for at least 48 h prior to the experiment, and were instructed to maintain a normal sleep pattern for 1 week. All participants slept for 7–9 h per day between 10:00 pm and 9:00 am, and their sleep routines throughout the study period were confirmed using sleep diaries. All participants provided written informed consent and received monetary compensation upon the completion of the experiment. This study was approved by the Ethics Committee of the Fourth Military Medical University.

### 2.2. Experimental design

**Figure 1:**
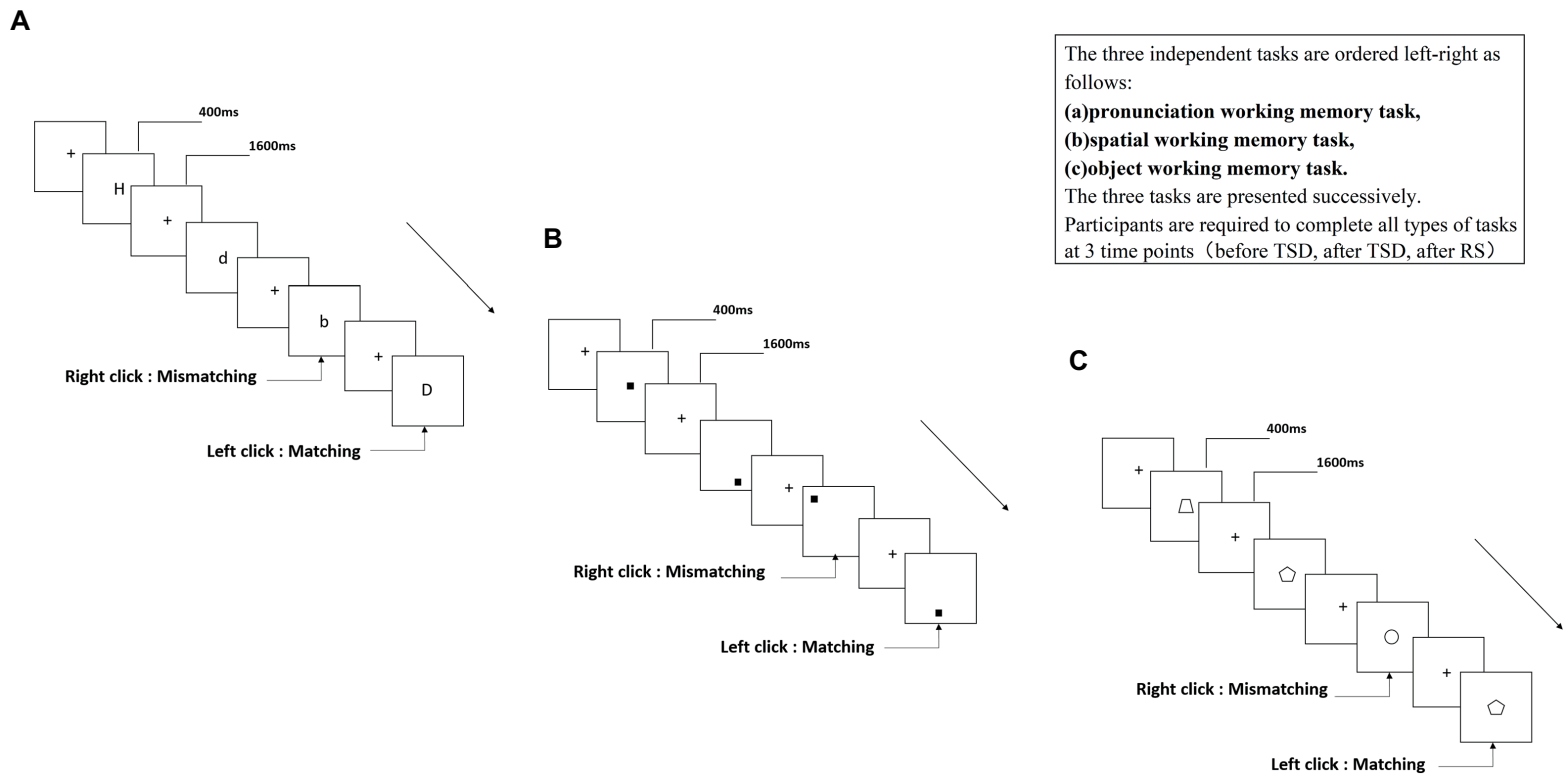
Schematic diagram of the working memory task.

In the present study, we used three tasks: a 2-back pronunciation working memory (PWM) task (Figure 1A), a 2-back spatial working memory (SWM) task (Figure 1B), and a 2-back object working memory (OWM) task (Figure 1C). The stimuli for these tasks included 15 case-insensitive English letters (excluding similar letters), small black squares, and 12 geometric figures. Each task lasted approximately 5 min and included 122 trials. Target stimuli were presented for 400 ms, with an inter-stimulus interval of 1,600 ms. Participants were asked to match the current stimulus with the stimulus presented two trials earlier, and were instructed to press the left mouse button for matching stimuli and the right mouse button for mismatching stimuli. The matching and mismatching stimuli were presented in a pseudo-random order in a 1:1 ratio.

According to the kind of stimulus material, working memory can be divided into the phonetic, spatial, and object types. To perform a more thorough investigation than past publications on the effects of sleep deprivation and restorative sleep on working memory, this study examined three types of working memory tasks. The tasks were independent of each other, and the participants exercised to eliminate the connection effect before engaging in the formal experiment.

### 2.3. Experimental procedures

**Figure 2:**
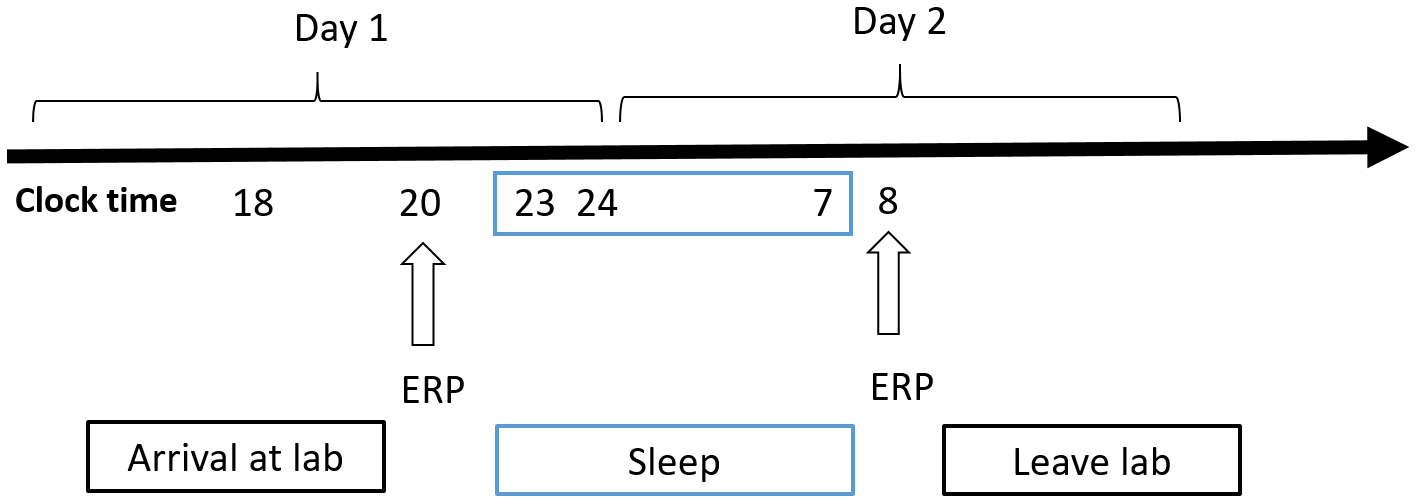
Experimental design for the nocturnal sleep (NS) group. Participants completed the tasks twice: before and after 8 h of sleep in the laboratory. Electroencephalographic data were recorded simultaneously. The arrows indicate various time points during the 2-back working memory task.

**Figure 3:**
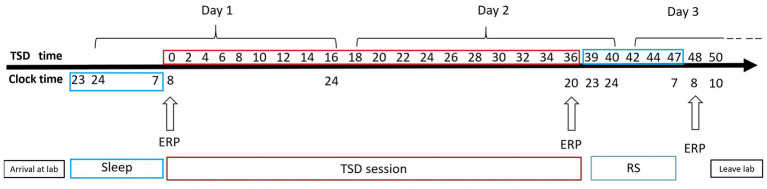
Experimental design for the sleep deprivation (SD) group. After 8 h of sleep in our laboratory, participants underwent 36 h of total sleep deprivation (TSD), followed by 8 h of recovery sleep (RS). Electroencephalographic data were recorded simultaneously. The arrows indicate various time points during the 2-back working memory task.

A mixed experimental design was used. Prior to testing, all participants practiced the experimental tasks until they reached an accuracy of >90% (to exclude the influence of practice). All the participants visited the laboratory once. The NS group arrived at the laboratory at 6:00 p.m. on the day of the experiment and performed the first task at 8:00 p.m. (baseline state; NS-BS). Following this, the participants slept in the laboratory that night (ensuring a sleep time of at least 8 h), woke up the next day at 7:00 am, and performed the second task at 8:00 am (0 h sleep deprivation; NS-SD0) with EEG data being recorded simultaneously (Figure 2). The SD group slept in the laboratory the night prior to completing the experimental tasks, and all participants were instructed to sleep from 11:00 pm to 7:00 am to ensure a sleep time of at least 8 h. The SD group underwent 36 h of SD followed by 8 h of RS (Figure 3). TSD was initiated at 8:00 am the following morning. The 2-back working memory tasks were performed both before and after TSD and 8 h after RS, with EEG data being recorded simultaneously.

Sleep inertia can be observed after waking up from complete and habitual night sleep and seems to be a common step in the sleep–wake transition process. Sleep inertia tends to be exacerbated by prior sleep loss or extended wakefulness prior to a sleep episode (Miccoli et al., 2008; Hilditch et al., 2017). To avoid sleep inertia, RS was implemented from 11:00 p.m. to 7:00 a.m., and a third set of EEG data was acquired at 8:00 a.m. on the third day (Dinges, 1990). Previous research has demonstrated that individual performance is relatively impaired within 5 min of awakening and gradually returns to normal over a period of 15–30 min (Dinges, 1986). Before TSD and 8 h after RS, the three types of working memory tasks were performed from 8:00 a.m. to 8:30 a.m. After TSD, the three types of working memory tasks were performed from 20:00 to 20:30. Two participants completed the experiment simultaneously. Two medical workers and one researcher were present throughout the TSD period to prevent the participants from sleeping or napping. During the experiment, participants were allowed to eat, drink, and perform light physical activities, but were not allowed to engage in strenuous exercise or ingest caffeine, alcohol, or tea. The illumination in the laboratory was set to 100 lx with a normal fluorescent lamp.

### 2.4. EEG recordings

EEG data were acquired in a dark, sound-proof, and electronically shielded EEG laboratory using 64-electrode caps. Stimuli were generated and presented using the Stim-2 software (NeuroScan Inc., United States). The electrodes were arranged in accordance with the international 10–20 system. Horizontal and vertical electrooculograms were recorded during EEG acquisition, and the bilateral mastoid process was used as the reference electrode. The recordings were performed at 1,000 Hz, and the channel impedance was maintained at below 5 kΩ.

### 2.5. Data analysis

We analyzed various behavioral parameters, including mean reaction time, accuracy, and the number of correct responses per unit time (number of correct responses per unit time = correct ratio × 1,000/correct time).

In this study, the EEG amplitude represented brain potential intensities. The amplitude size is closely related to the number of neurons involved in synchronous firing, as well as the arrangement direction of the neurons. Latency provided a measure the time interval from stimulus presentation to the peak amplitude value for each condition (Kiesel et al., 2008). ERP data could not be recorded in three cases due to technical issues. Data from these three participants were excluded during post-processing (NS group: 19; SD group: 20).

EEG data were pre-processed using the SCAN 4.3 software (Neuroscan, Inc., United States), following which ocular artifacts were removed *via* regression analysis. The data were bandpass filtered at 0.05–30 Hz (frequency slope: 24 dB/oct) and were divided into 900-ms epochs (−100 ms to 800 ms). A period of 100 ms prior to stimulation was included for baseline correction. Trials in which the voltage exceeded ±100 μV were excluded. Non-physiological artifacts mainly included those generated from the contact between the electrode and the scalp to the device or to the environment (i.e., the environment around the device or the device in the participant). Generally, non-physiological artifacts display various waveforms, which may preclude data interpretation in severe cases (Maddirala and Shaik, 2016). The mean number of accepted trials was 92.4 ± 17.46 (NS: 95.6 ± 7.85 vs. SD: 89.2 ± 27.07, *p* > 0.05). As guided by previous studies, P3 (250–450 ms) and N2 components (150–350 ms) were analyzed for the following channels: F3, Fz, F4, C3, Cz, C4, P3, Pz, and P4 (Zhang et al., 2019; Peng et al., 2020).

Repeated-measures analysis of variance (ANOVA) was used to analyze both the behavioral data and ERP findings using SPSS (version 22.0, IBM Corp., United States).

### 2.6. Statistical analysis of data from the NS and SD groups

For ERP analyses, we assessed the main and interaction effects of the groups (NS and SD), sleep states (NS-BS and NS-SD0; SD-SD0 and SD-SD36), tasks (PWM, SWM, and OWM), regions (frontal, central, and parietal), and sites (left, middle, and right). Behavioral data were compared between the two groups (NS and RS), two sleep states (no-sleep deprived: NS-BS and NS-SD0; sleep deprived: SD-SD0 and SD-SD36), and three tasks (PWM, SWM, and OWM). Greenhouse–Geisser corrections for non-sphericity and *post hoc* tests with Bonferroni correction were performed. The results are presented as the mean and standard deviation.

### 2.7. Statistical analysis of data from the SD groups

To analyze ERP data, we assessed the main and interaction effects of sleep states (SD-SD0, SD-SD36, and RS-8 h), tasks (PWM, SWM, and OWM), regions (frontal, central, and parietal), and sites (left, middle, and right). We analyzed the same behavioral parameters as those mentioned above, and compared behavioral data between the three sleep states (RS-SD0, RS-SD36, and RS-8 h) and three tasks (PWM, SWM, and OWM). Greenhouse–Geisser corrections for non-sphericity and *post hoc* tests with Bonferroni correction were performed. The results are presented as the mean and standard deviation.

## 3. Results

### 3.1. Results of NS vs. SD comparisons

#### 3.1.1. Behavioral performance

**Figure 4:**
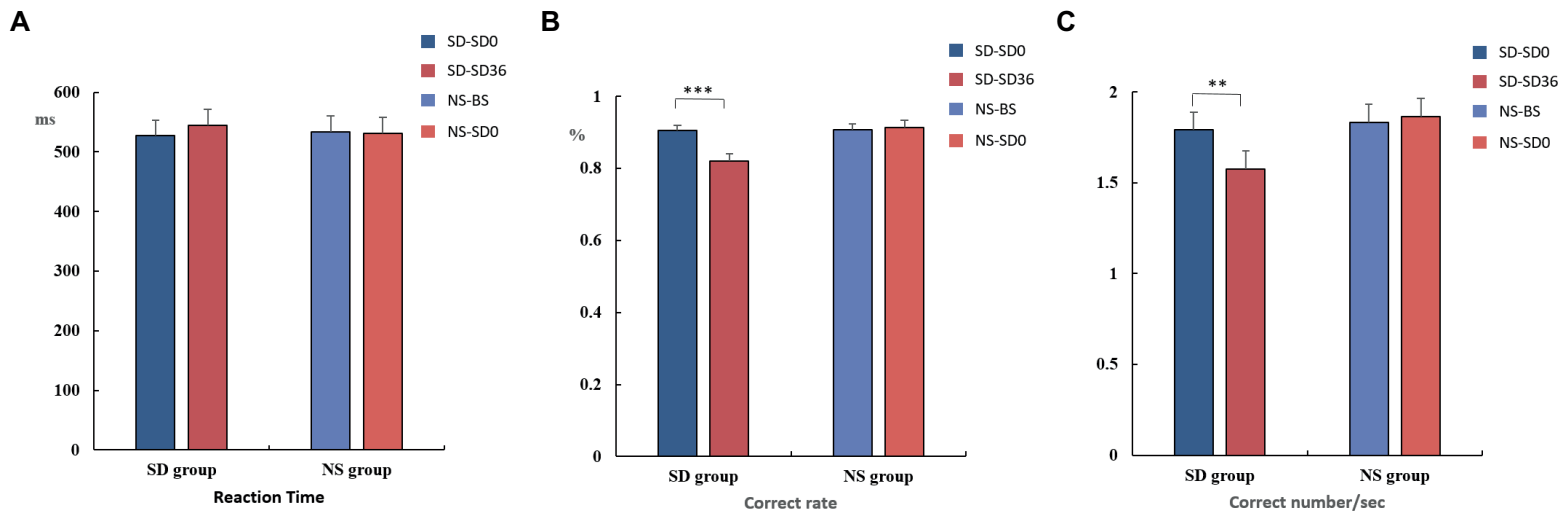
Reaction time, rate of correct responses, and number of correct responses per unit time (mean ± standard deviation). SD, sleep deprivation; BS, baseline; NS, nocturnal sleep. *p < 0.05, **p < 0.01, ***p < 0.001.

The mean reaction time, accuracy, and number of correct responses per unit time are presented in Tables 1, 2. For accuracy (*F*_(1, 37)_ = 16.420, *p* < 0.001, *η*^2^*_p_* = 0.307) and number of correct responses per unit time (*F*_(1, 37)_ = 4.869, *p* = 0.034, *η*^2^*_p_* = 0.116), there were significant interaction effects between group and sleep state. These results suggested that in the SD group, accuracy (*p* < 0.001) and the number of correct responses per unit of time (*p* = 0.009) decreased significantly after 36 h of TSD. However, there was no statistically significant differences between the two states in the NS group (Figure 4). No other main or interaction effects were statistically significant.

**Table 1:** Behavioral performance (mean ± standard deviation) in the three types of 2-back tasks in the sleep deprivation (SD) group.

	SD-0 h			SD-36 h			RS-8 h		
	PWM	SWM	OWM	PWM	SWM	OWM	PWM	SWM	OWM
Mean reaction time (ms)	539.94 (101.20)	520.16 (91.58)	522.19 (88.44)	561.88 (104.23)	527.46 (94.15)	543.85 (107.88)	528.09 (66.34)	498.80 (84.98)	526.07 (80.19)
Correct rate (%)	0.89 (0.10)	0.95 (0.04)	0.88 (0.09)	0.82 (0.13)	0.85 (0.12)	0.79 (0.13)	0.88 (0.08)	0.93 (0.04)	0.87 (0.07)
Correct number/s	1.73 (0.45)	1.88 (0.35)	1.76 (0.44)	1.53 (0.40)	1.68 (0.41)	1.53 (0.45)	1.70 (0.30)	1.93 (0.36)	1.69 (0.34)

PWM, pronunciation working memory; SWM, spatial working memory; OWM, object working memory.

**Table 2:** Behavioral performance (mean ± standard deviation) in the three types of 2-back tasks in the nocturnal sleep (NS) group.

	BS			SD-0 h		
	PWM	SWM	OWM	PWM	SWM	OWM
Mean reaction time (ms)	562.51 (163.77)	518.58 (137.09)	519.83 (117.88)	557.28 (165.27)	495.90 (136.18)	538.57 (134.62)
Correct rate (%)	0.90 (0.10)	0.93 (0.05)	0.90 (0.07)	0.90 (0.08)	0.94 (0.04)	0.90 (0.07)
Correct number/s	1.74 (0.53)	1.92 (0.50)	1.83 (0.48)	1.76 (0.54)	2.04 (0.55)	1.79 (0.48)

PWM, pronunciation working memory; SWM, spatial working memory; OWM, object working memory.

#### 3.1.2. The N2 component

The descriptive statistics for the N2 component of the NS group are presented in Tables 3, 4. N2 latency (*F*_(1, 37)_ = 4.426, *p* = 0.042, η^2^*_p_* = 0.107) was affected by significant interaction effects between group and sleep state. N2 latency was significantly prolonged after a 36 h TSD in the SD group (*p* = 0.002). However, there was no difference in N2 latency between the two sleep states in the NS group (*p* = 0.085). For N2 amplitude, there were no significant interaction effects between the groups and sleep states (*F*_(1, 37)_ = 0.004, *p* = 0.947, *η*^2^*_p_* < 0.001). No other main or interaction effects were statistically significant.

**Table 3:** Grand-average peak latency of the N2 and P3 components for correct responses across multiple electrode sites at baseline and after 0 h of sleep deprivation (SD0) in the nocturnal sleep (NS) group.

		Baseline		SD-0 h	
		N2	P3	N2	P3
F3	M (SD)	238.94 (29.63)	363.22 (33.47)	241.55 (32.17)	363.44 (30.42)
Fz	M (SD)	237.69 (29.24)	359.19 (33.29)	242.07 (31.22)	358.87 (33.56)
F4	M (SD)	232.58 (27.75)	360.64 (31.81)	230.14 (28.15)	359.71 (32.25)
C3	M (SD)	228.34 (26.27)	353.49 (31.16)	228.347 (30.14)	358.41 (32.03)
Cz	M (SD)	222.83 (23.89)	351.83 (30.23)	227.66 (27.92)	350.44 (32.31)
C4	M (SD)	220.66 (24.04)	359.01 (28.57)	218.66 (26.21)	353.82 (29.44)
P3	M (SD)	206.81 (31.96)	346.72 (28.67)	207.25 (29.44)	347.09 (38.32)
Pz	M (SD)	207.52 (23.71)	345.30 (31.13)	214.69 (29.20)	347.03 (36.55)
P4	M (SD)	212.61 (32.96)	322.81 (31.84)	205.50 (33.84)	332.04 (32.02)

**Table 4:** Grand-average peak amplitude of the N2 and P3 components for correct responses across multiple electrode sites at baseline and after 0 h of sleep deprivation (SD0) in the nocturnal sleep (NS) group.

		Baseline		SD-0 h	
		N2	P3	N2	P3
F3	M (SD)	−2.90 (3.94)	6.97 (2.37)	−2.11 (3.80)	5.76 (2.89)
Fz	M (SD)	−3.22 (4.40)	7.79 (2.36)	−2.22 (3.97)	6.63 (3.48)
F4	M (SD)	−2.45 (4.05)	8.13 (2.60)	−1.54 (3.75)	7.40 (3.24)
C3	M (SD)	−2.04 (3.61)	7.73 (2.06)	−1.94 (2.86)	6.91 (2.97)
Cz	M (SD)	−1.66 (4.24)	9.38 (2.26)	−1.95 (3.52)	8.02 (3.41)
C4	M (SD)	−1.02 (3.50)	9.30 (2.34)	−1.45 (3.13)	8.17 (3.26)
P3	M (SD)	−2.89 (5.50)	7.76 (2.49)	−2.20 (4.84)	7.80 (3.01)
Pz	M (SD)	−1.08 (3.59)	8.51 (2.68)	−1.76 (3.63)	8.21 (2.73)
P4	M (SD)	−1.17 (4.00)	7.02 (2.43)	−2.34 (5.35)	7.74 (3.07)

#### 3.1.3. The P3 component

**Figure 5:**
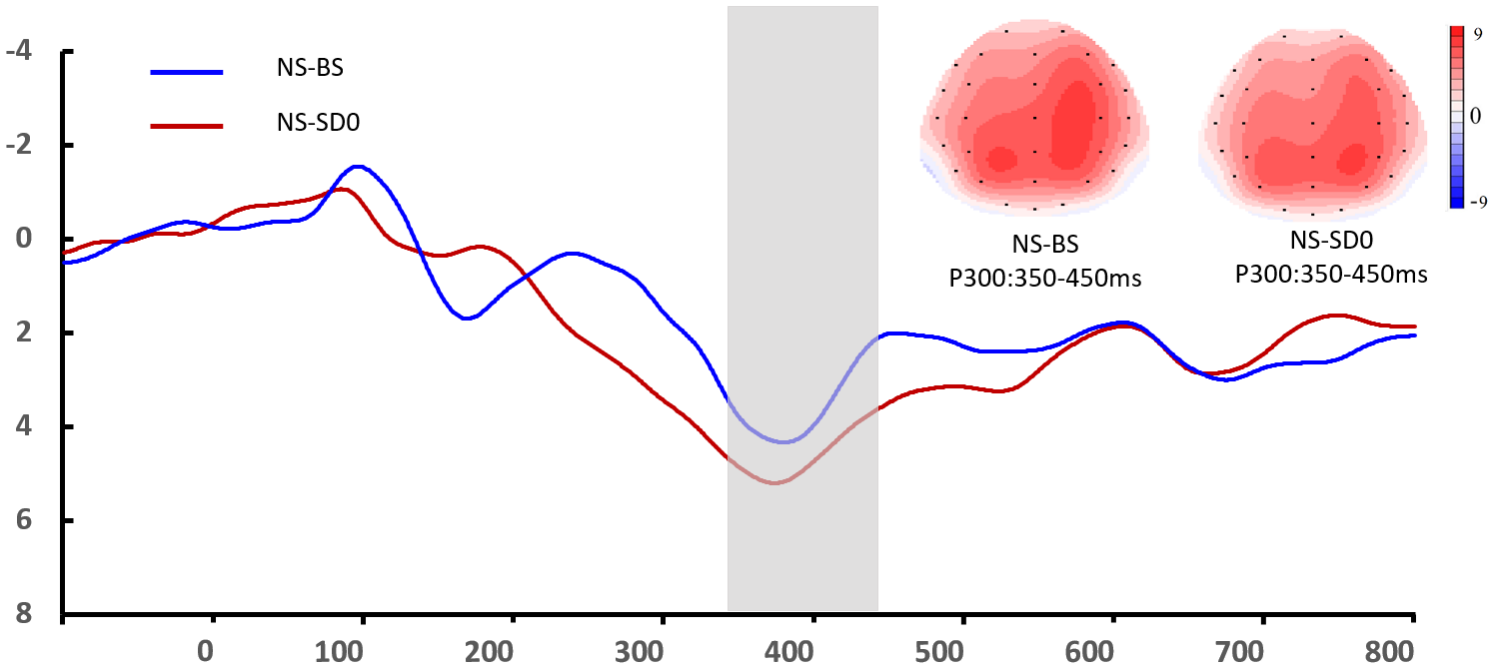
Grand mean amplitude of the P3 component in the nocturnal sleep (NS) group at the baseline state (BS) and after 0 h of sleep deprivation (SD0). Averaged data from the F3, Fz, F4, C3, Cz, C4, P3, Pz, and P4 electrodes are shown. The topographies correspond to average activity in the time windows (350–450 ms, indicated by the gray bar) around the local peaks.

**Figure 6:**
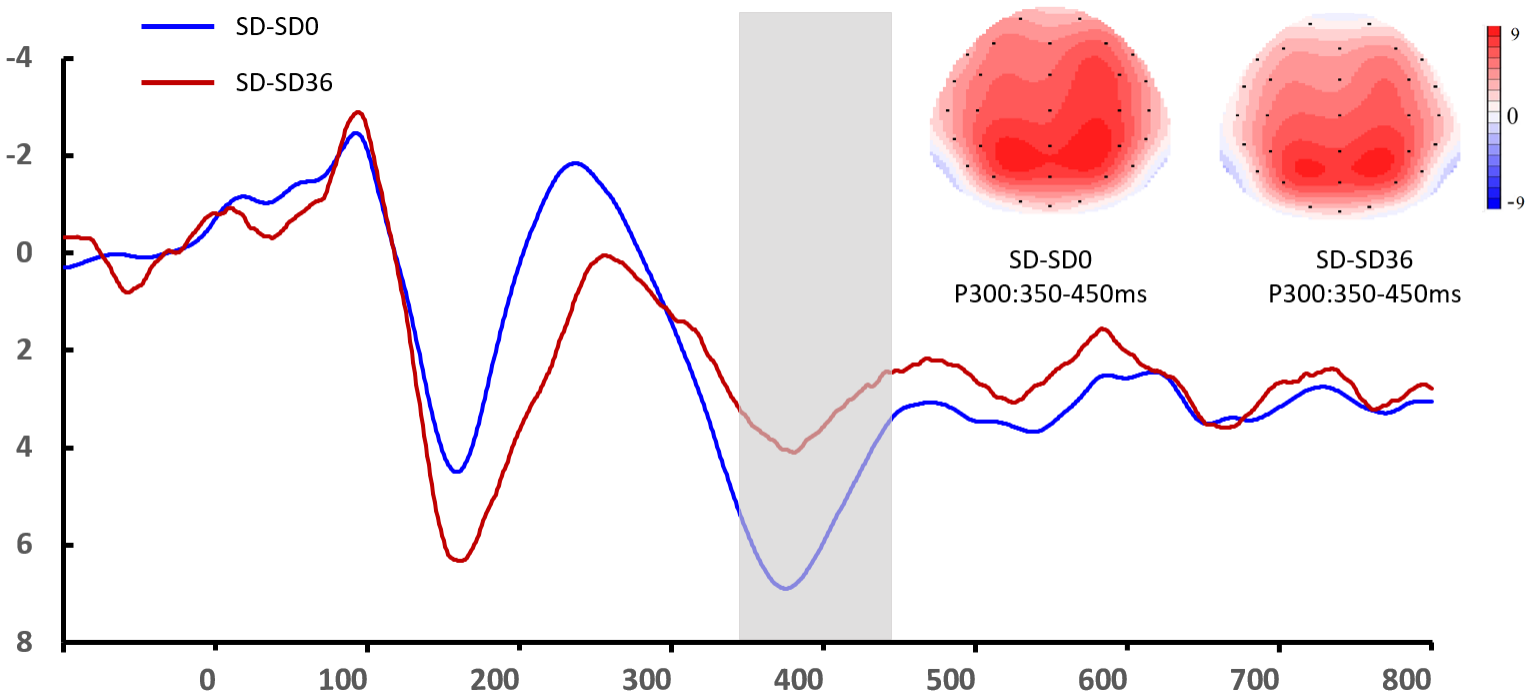
Grand mean amplitude of the P3 component in the sleep deprivation (SD) group after 0 h of sleep deprivation (SD0) and 36 h of sleep deprivation (SD36). Averaged data from the F3, Fz, F4, C3, Cz, C4, P3, Pz, and P4 electrodes are shown. The topographies correspond to average activity in the time windows (350–450 ms, indicated by the gray bar) around the local peaks.

The descriptive statistics for the P3 component of the NS group are presented in Tables 3, 4. For P3 latency (*F*_(1, 37)_ = 0.002, *p* = 0.962, *η*^2^*_p_* < 0.001) and P3 amplitude (*F*_(1, 37)_ = 0.902, *p* = 0.348, *η*^2^*_p_* = 0.024), there were no significant interaction effects between group and sleep state. However, a simple effect analysis revealed that before sleep deprivation, the P3 amplitude was significantly lower in the SD group (*p* = 0.013). The average amplitudes and latencies of P3 as elicited by the nine electrode sites are presented in Figures 5, 6. No other main or interaction effects were statistically significant.

### 3.2. Results of SD vs. RS comparisons

#### 3.2.1. Behavioral performance

**Figure 7:**
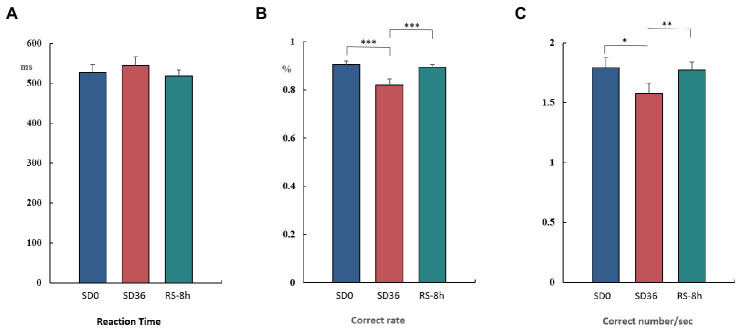
Reaction time, rate of correct responses, and number of correct responses per unit time in the three sleep states (mean ± standard deviation). SD: sleep deprivation; RS: recovery sleep. *p < 0.05, **p < 0.01, ***p < 0.001.

The results of the behavioral experiments are presented in Table 1. The mean reaction time tended to be longer after the 36 h TSD and tended to be shorter after 8 h of RS, although the differences were not significant (*F*_(2, 38)_ = 1.07, *p* = 0.353, *η*^2^*_p_* = 0.053). For accuracy (*F*_(2, 38)_ = 17.023, *p* < 0.001, *η*^2^*_p_* = 0.473) and the number of correct responses per unit time (*F*_(2, 38)_ = 5.893, *p* = 0.006, *η*^2^*_p_* = 0.237), there was a significant main effect of sleep state. The accuracy and number of correct responses per unit time were significantly decreased after the 36 h TSD (accuracy: *p*< 0.001; number of correct responses per unit time: *p* = 0.02) and were restored after the 8 h RS (accuracy: *p* < 0.001; number of correct responses per unit time: *p* = 0.006) (Figure 7). No other main or interaction effects were statistically significant.

#### 3.2.2. The N2 component

The descriptive statistics for the N2 component of the RS group are presented in Tables 5, 6. For the N2 latency, there was a significant main effect of sleep state (*F*_(2, 38)_ = 4.511, *p* = 0.017, *η*^2^*_p_* = 0.192). After the 36 h TSD, the N2 latency (*p* = 0.01) increased significantly and showed a decreasing trend after the 8 h RS (*p* = 0.11). There was no main effect of sleep state on the amplitude of N2 (*F*_(2, 38)_ = 0.465, *p* = 0.631, *η*^2^*_p_* = 0.024). No other main or interaction effects were statistically significant.

**Table 5:** Grand-average peak latency of the N2 and P3 components for correct responses across multiple electrode sites in three sleep states in the sleep deprivation (SD) group.

		SD-0 h		SD-36 h		RS-8 h	
		N2	P3	N2	P3	N2	P3
F3	M (SD)	232.57 (36.77)	372.41 (33.91)	250.74 (49.32)	374.85 (33.49)	243.72 (39.16)	372.36 (36.55)
Fz	M (SD)	235.89 (38.35)	370.90 (34.97)	247.39 (44.22)	378.39 (32.22)	242.68 (36.11)	369.26 (35.48)
F4	M (SD)	229.21 (36.35)	371.33 (31.72)	248.93 (48.36)	377.63 (33.64)	238.15 (39.51)	370.58 (33.27)
C3	M (SD)	217.58 (33.88)	367.87 (33.40)	231.75 (46.67)	369.35 (29.87)	225.80 (39.55)	363.95 (38.51)
Cz	M (SD)	224.36 (36.51)	361.43 (34.43)	229.79 (45.19)	370.04 (34.69)	230.90 (40.53)	357.77 (35.88)
C4	M (SD)	215.79 (35.35)	368.32 (27.19)	230.50 (44.68)	368.90 (34.81)	229.42 (39.82)	360.26 (37.63)
P3	M (SD)	207.55 (45.76)	359.62 (34.36)	209.64 (34.93)	362.05 (40.21)	211.55 (39.28)	352.95 (36.65)
Pz	M (SD)	206.46 (31.04)	353.29 (36.24)	223.56 (43.61)	352.26 (37.77)	211.94 (34.55)	348.34 (41.02)
P4	M (SD)	209.79 (46.66)	334.74 (39.05)	222.84 (45.27)	337.95 (45.44)	212.25 (45.42)	338.58 (45.31)

**Table 6:** Grand-average peak amplitude of the N2 and P3 components for correct responses across multiple electrode sites in three sleep states in the sleep deprivation (SD) group.

		SD-0 h		SD-36 h		RS-8 h	
		N2	P3	N2	P3	N2	P3
F3	M (SD)	−2.50 (4.17)	7.80 (3.35)	−3.27 (4.93)	6.33 (3.21)	−3.35 (2.92)	7.74 (4.33)
Fz	M (SD)	−2.72 (3.95)	8.31 (3.44)	−3.48 (5.87)	7.85 (3.87)	−4.03 (3.44)	8.47 (5.12)
F4	M (SD)	−1.88 (3.66)	8.93 (3.38)	−2.69 (4.58)	7.11 (3.85)	−3.06 (3.42)	9.09 (4.67)
C3	M (SD)	−2.04 (4.03)	8.49 (3.39)	−2.09 (4.75)	6.88 (3.56)	−2.30 (2.62)	8.92 (4.52)
Cz	M (SD)	−1.93 (4.51)	9.64 (3.65)	−1.68 (5.36)	8.14 (3.28)	−2.20 (3.69)	10.36 (5.20)
C4	M (SD)	−1.36 (3.63)	9.05 (3.03)	−0.93 (4.29)	7.58 (3.38)	−1.08 (3.03)	10.35 (4.46)
P3	M (SD)	−2.55 (5.08)	8.51 (3.82)	−2.53 (4.93)	7.27 (3.55)	−2.81 (4.87)	8.71 (4.19)
Pz	M (SD)	−0.68 (4.17)	9.20 (4.07)	−0.91 (4.21)	8.36 (3.68)	−1.05 (4.13)	9.96 (4.22)
P4	M (SD)	−1.65 (4.03)	8.00 (3.41)	−0.93 (4.91)	7.79 (4.41)	−1.60 (3.99)	8.30 (3.77)

#### 3.2.3. The P3 component

**Figure 8:**
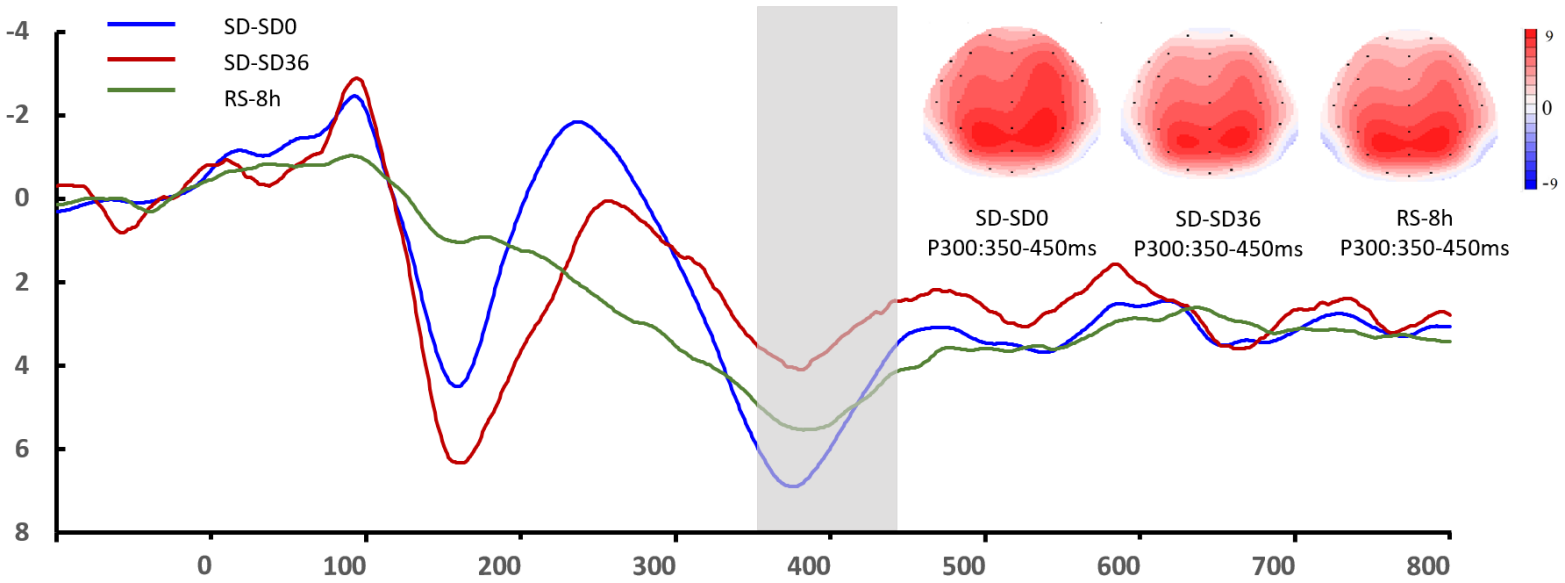
Grand mean amplitude of the P3 component in the sleep deprivation (SD) group after 0 h of sleep deprivation (SD0), 36 h of sleep deprivation (SD36), and 8 h of recovery sleep (RS-8 h). Averaged data from F3, Fz, F4, C3, Cz, C4, P3, Pz, and P4 electrodes are shown. The topographies correspond to average activity in the time windows (350–450 ms, indicated by the gray bar) around the local peaks.

The descriptive statistics for the P3 component of the RS group are presented in Tables 5, 6. For P3 amplitude, there was a significant main effect of sleep state (*F*_(1.52, 28.95)_ = 4.948, *p* = 0.012, η^2^_P_ = 0.207). After the 36 h TSD, the P3 amplitude (*p* = 0.003) decreased significantly and was restored after the 8 h RS (*p* = 0.015). We also observed a significant interaction effect of sleep state and region on the P3 amplitude (*F*_(1.87, 35.55)_ = 2.489, *p* = 0.050, η^2^_P_ = 0.116). During the three sleep states, the fluctuations in P3 were mainly focused in frontal and central regions (Figure 8). For latency of P3, the main effect of and sleep state was not significant (*F*_(2，38)_ = 11.921, *p* = 0.160, *η*^2^*_p_* = 0.092). No other main or interaction effect was statistically significant.

## 4. Discussion

In the present study, we analyzed ERPs to investigate the effects of 8 h of RS on working memory impairment induced by 36 h of TSD. The results of our previous study had revealed that TSD significantly impairs the accuracy of 2-back working memory tasks (Peng et al., 2020). The current behavioral findings demonstrate that 8 h of RS can improve both the accuracy of responses and the number of correct responses per unit time in such tasks. Changes in behavioral indicators effectively reflect the improvement in working memory ability, which may also be related to the recovery of vigilance and disposable attentional resources (Doty et al., 2017). Although a constant cognitive load (2-back) was utilized in the present study, our results indicated that working memory ability improved after 8 h of RS when compared with the performance observed after 36 h of TSD without RS. Moreover, despite there being no marked improvements in reaction time after 8 h of RS, the number of correct responses per unit time increased significantly. Changes in accuracy rates can also influence response times; as such, there may be situations where participants sacrifice accuracy to reduce reaction time (de Bruijn et al., 2020). The number of correct responses per unit of time combines reaction time and accuracy and more accurately reflects an individual’s working memory ability and level of cognitive control.

Notably, 8 h of RS after TSD also induced a significant decrease in N2 latency and a significant increase in P3 amplitude. The N2 component is considered to reflect an individual’s mental state and level of attention (Schacht et al., 2008), whereas the P3 component is thought to be involved in the decision-making process during cognitive matching tasks (Gosselin et al., 2005). Increases in P3 latency and decreases in P3 amplitude are associated with prolonged wakefulness (Panjwani et al., 2010).In addition, several studies have reported a decrease in reaction time and sustained attention following SD (Rupp et al., 2009; Chua et al., 2014). Thus, our findings are in accordance with previous results and support the notion that 8 h of RS can improve performance and alertness (Zhang et al., 2014). The restoration of attention and alertness following RS may have enabled participants to allocate more attentional resources to working memory tasks, thereby attenuating TSD-induced impairments (Donchin and Fabiani, 1991). Previous studies have also indicated that compared with drowsiness, SD is associated with more pronounced decreases in the activation of the frontoparietal network (which in involved in working memory) (Almklov et al., 2015). Furthermore, SD can reduce metabolic activity in regions associated with information processing and executive control (Choo et al., 2005), whereas RS can restore the overall network organization following TSD (Jiang et al., 2018).

Sustained attention and alertness are essential for the performance of daily activities. Based on the observed changes in the N2 and P3 components (i.e., increased amplitude and decreased latency) after 8 h of RS, we speculated that RS can effectively attenuate impairments in attention and alertness, thus influencing the information integration process. The deterioration of sustained attention seems to be a long-lasting negative effect of SD (de Bruin et al., 2017; Lowe et al., 2017), and is likely caused by decreased arousal of the central nervous system (CNS) (Schneider and Fisk, 1984; Cote et al., 2009). In contrast, more automatic or bottom-up processes appear to be less affected by changes in CNS arousal (Schneider and Fisk, 1984). Therefore, improvements in sustained attention are likely to occur earlier given the greater sensitivity of sustained attention to SD. The completion of a cognitive task usually requires the joint participation of several psychological processes, including early sensory perception, alertness, basic attentional mechanisms, working memory, and decision making. The P3 component appears relatively late, suggesting that it is more reflective of conscious participation and likely involves top-down cognitive control (Kusztor et al., 2019). The observed increases in amplitude and decreases in latency also suggest that 8 h of RS improves the ability to integrate dynamic information during working memory tasks. Communication between the hippocampus and prefrontal areas is vital for the optimal redistribution of temporal memory traces to more resident cortical storage. Therefore, interrupting this communication may impair an individual’s ability to form a new memory. In this regard, Chai et al. recently reported that RS re-normalizes hippocampal connections (Chai et al., 2020).

Normal sleep is divided into two phases: rapid eye movement (REM) and slow-wave sleep (SWS). Deep sleep during the N3 stage of SWS is particularly important for restoring mental and physical energy. Following SD, we observed compensatory responses during the restorative sleep stages. Interestingly, the intensity (rather than the duration) of sleep influences the recovery of function following SD. Sleep intensity during SWS is regarded as an indicator of homeostatic sleep pressure (Borbély, 1982; Hennecke et al., 2019). After one night of SD, less than 10 h of RS can sufficiently reduce the level of sleep stress to that observed at the end of a typical 8 h period of normal sleep (Daan et al., 1984; Achermann and Borbély, 1994). Nevertheless, further increases in the duration of SWS have been observed on the second night of recovery (Carskadon and Dement, 1985). RS exhibits characteristics distinct from those of normal sleep, including a decrease in sleep-onset latency. Key changes have also been observed during the N2 and N3 stages. In addition, longer periods of RS result in a sleep stage distribution similar to that of normal sleep (Hennecke et al., 2019). Therefore, individuals in the RS group experienced increases in the proportion of SWS during RS relative to the amount observed during normal sleep. It is possible that SD-induced impairments in working memory function are specifically attenuated during SWS.

The behavioral and EEG data obtained in this study support our hypothesis that 8 h of RS can attenuate impairments in working memory caused by 36 h of TSD. However, we did not observe significant changes in all the indicators identified in our previous study (Peng et al., 2020). Insufficient sleep may lead individuals to provide conservative estimates of their performance, which may increase the likelihood of compensatory behaviors and protect against the negative consequences of SD (Boardman et al., 2018). Therefore, the results of this study should be interpreted with caution. Previous research has demonstrated that simple cognitive responses are less affected by SD and can be easily recovered following RS, whereas impairments in higher-level cognitive functions are less easily reversed (Nilsson et al., 2005; Skurvydas et al., 2020). Improvements in cognitive function following RS are mainly reflected by changes in alertness and sustained attention, which allow participants to allocate more attentional resources to the current task (Jin et al., 2015). Nonetheless, further studies are required to elucidate the mechanisms by which RS restores cognitive function after TSD. The main goal of this study was to further explore the recovery effect of 8 h of restorative sleep on impaired cognitive ability based on previous findings (i.e., how sleep deprivation impairs working memory or other cognitive functions). In addition, we also included a blank control group, unlike most prior studies.

The present study has some limitations. We did not assess working memory performance using tasks of varying difficulty, which limits our ability to infer how changes in workload impact the restorative effect of RS. In addition, our study included only male volunteers, and caution should be exercised when attempting to extend our findings to female individuals. All participants in this study had good sleep quality; however, the impact of sleep deprivation can differ between the normal population and people with insomnia or rhythm disorders. Rhythm disorders can cause changes in an individual’s melatonin secretion cycle, leading to specific deficits in the neurophysiological activity in the attention domain (Gumenyuk et al., 2014). Therefore, our inferences may be limited to an optimally sleeping population. Considering the number of participants in our study and some non-significant findings related to EEG indicators, further studies are required to determine whether 8 h of RS can restore cognitive function to baseline levels. In future studies, we plan to use analytical methods based on the power spectrum. Multimodal studies involving brain network analyses of EEG and imaging data can also help further explain our results. We did not use sleep monitoring technology to investigate whether RS induces specific alterations in sleep structure, necessitating further studies in this regard. Finally, circadian biorhythms are known to affect behavioral performance (Montplaisir, 1981), and their effects should also be considered in future studies.

Our results align with those of previous studies, suggesting that 8 h of RS can partially attenuate the deleterious effects of TSD on working memory. This study provides experimental evidence of the recovery of cognitive function after acute sleep loss. RS has potential value as an applied non-pharmacological strategy to alleviate the effects of sleep deprivation. For example, failure to maintain a high level of alertness may lead to serious consequences during military missions. The widespread use of high-tech equipment demands high levels of cognitive ability and brain function, and sleep deprivation may become more prominent under high-tech warfare conditions in future. Therefore, strengthening research on sleep deprivation and providing effective medical support under continuous combat conditions is of great significance to the success of military endeavors.

## 5. Conclusion

In summary, our results suggest that RS may exert its effects by improving alertness and sustained attention in sleep-deprived individuals. Because SWS dominates the sleep period during RS, these restorative effects are likely to occur during SWS. However, RS had limited effects in the present study, and further studies are required to determine whether 8 h of RS can restore cognitive function to baseline levels.

## Data availability statement

The raw data supporting the conclusions of this article will be made available by the authors, without undue reservation.

## Ethics statement

The studies involving human participants were reviewed and approved by the Fourth Military Medical University. The patients/participants provided their written informed consent to participate in this study.

## Author contributions

YS, ZP, and YH designed the study. ZP produced the results and wrote the manuscript. LX, HW, SW, and TS contributed data collection and analysis. YY and YS were the guarantors of this study. All authors contributed to the article and approved the submitted version.

## Funding

This research was supported by the National Science Foundation of China: 211CXGCM113040301.

## Conflict of interest

The authors declare that the research was conducted in the absence of any commercial or financial relationships that could be construed as a potential conflict of interest.

## Publisher’s note

All claims expressed in this article are solely those of the authors and do not necessarily represent those of their affiliated organizations, or those of the publisher, the editors and the reviewers. Any product that may be evaluated in this article, or claim that may be made by its manufacturer, is not guaranteed or endorsed by the publisher.
